# Supplementary material for: Genome-wide association study of actinic keratosis identifies new susceptibility loci implicated in pigmentation and immune regulation pathways
Source: Commun Biol. 2022 Apr 21;5:386. doi: 10.1038/s42003-022-03301-3 (PMC9023580; doi:10.1038/s42003-022-03301-3)
Supplement: Supplementary file 2 — Supplementary Information [file 42003_2022_3301_MOESM2_ESM.pdf]

Supplementary Table 1. Lead genome-wide significant SNP for each independent locus identified among those without cSCC history (n=55,989) in the GERA discovery cohort

SNP	Locus	Gene[1]	Ref/Eff allele	GERA discovery cohort	
				OR (95% CI)	P-value[3]
rs62247035	3p13	*FOXP1*	G/A	1.08 (1.05, 1.12)	1.10E-06
rs16891982	5p13	*SLC45A2*	G/C	0.49 (0.44, 0.53)	7.50E-52
rs12203592	6p25	*IRF4*	C/T	1.48 (1.43, 1.54)	2.20E-86
rs9271377	6p21	*HLA-DQA1*	T/G	1.06 (1.03, 1.09)	3.40E-04
rs12350739	9p22	*BNC2*	A/G	0.87 (0.84, 0.90)	4.80E-18
rs1126809	11q14	*TYR*	G/A	1.09 (1.05, 1.13)	1.10E-06
rs12916300	15q13	*HERC2*	T/C	0.89 (0.86, 0.93)	7.90E-10
rs4268748	16q24	*DEF8*	T/C	1.24 (1.19, 1.28)	6.80E-29
rs35063026[2]	16q24	*SPATA33*	C/T	1.34 (1.27, 1.42)	3.40E-25
rs6059655	20q11	*RALY*	G/A	1.3 (1.23, 1.37)	2.10E-21
rs2425025	20q11	*MMP24*	A/G	1.3 (1.22, 1.38)	8.80E-16

1 Novel loci are underlined

2 Independently AK-associated SNP on conditional analysis

3 the P-value adjusted for Bonferroni correction was set as P<4.5x10-3 (0.05/11)

SNP: Single Nucleotide Polymorphism; Chr: Chromosome; Pos: Position; MAF: Minor Allele Frequency; Ref/Eff allele: Reference/Effect allele; OR: Odds Ratio; CI: Confidence Interval

Supplementary Table 2. VEGAS2-gene based analysis top results

Chr	Gene	nSNPs	Start	Stop	Pvalue	p_fdr_dh	TopSNP	TopSNP.pvalue
1	ANXA9	79	150944498	150978114	1.00E-06	4.80E-04	rs2305814	1.95E-06
3	MIR1284	27	71581120	71601240	1.00E-06	4.80E-04	rs35480566	5.45E-11
5	RXFP3	58	33926490	33949023	1.00E-06	4.80E-04	rs35395	1.46E-55
5	SLC45A2	95	33934720	33994780	1.00E-06	4.80E-04	rs16891982	8.43E-71
6	DUSP22	31	282056	361355	1.00E-06	4.80E-04	rs7745355	7.40E-06
6	EXOC2	815	475137	703141	1.00E-06	4.80E-04	rs12210050	2.14E-50
6	HLA-DQA1	852	32595182	32621429	1.00E-06	4.80E-04	rs4455710	3.77E-10
6	HLA-DRB1	834	32536546	32567613	1.00E-06	4.80E-04	rs116239728	4.47E-08
6	HLA-DRB5	470	32475153	32508006	1.00E-06	4.80E-04	rs80292027	1.64E-07
6	IRF4	117	381738	421443	1.00E-06	4.80E-04	rs12203592	1.97E-155
9	BNC2	578	16399500	16880786	1.00E-06	4.80E-04	rs2153271	3.50E-19
11	TYR	374	88901039	89038927	1.00E-06	4.80E-04	rs1126809	1.17E-17
15	HERC2	232	28346182	28577298	1.00E-06	4.80E-04	rs12916300	5.32E-15
16	AFG3L1P	144	90028987	90077195	1.00E-06	4.80E-04	rs35176381	1.37E-46
16	ANKRD11	413	89324028	89566969	1.00E-06	4.80E-04	rs74836424	2.13E-38
16	C16orf3	84	90085315	90106309	1.00E-06	4.80E-04	rs10431948	1.49E-23
16	CDK10	82	89743075	89772772	1.00E-06	4.80E-04	rs12922197	2.01E-41
16	CENPBD1	62	90026182	90049240	1.00E-06	4.80E-04	rs4268748	9.82E-56
16	CHMP1A	95	89700838	89734193	1.00E-06	4.80E-04	rs71396950	3.80E-50
16	CIITA	101	10961054	11028840	1.00E-06	4.80E-04	rs12931265	1.23E-07
16	CPNE7	7	89632175	89673654	1.00E-06	4.80E-04	rs34395984	2.95E-43
16	DBNDD1	128	90061272	90096539	1.00E-06	4.80E-04	rs35176381	1.37E-46
16	DEF8	118	90005138	90044468	1.00E-06	4.80E-04	rs4268748	9.82E-56
16	DPEP1	51	89669715	89714839	1.00E-06	4.80E-04	rs75923656	9.06E-44
16	FANCA	503	89793958	89893065	1.00E-06	4.80E-04	rs75570604	5.35E-49
16	GAS8	178	90076036	90121379	1.00E-06	4.80E-04	rs77381714	2.34E-42

16	LOC100287036	38	89377540	89401518	1.00E-06	4.80E-04	rs113955902	1.58E-28
16	LOC101927817	85	89487325	89530902	1.00E-06	4.80E-04	rs74836424	2.13E-38
16	MC1R	43	89974286	89997385	1.00E-06	4.80E-04	rs1805007	1.12E-46
16	PRDM7	102	90112973	90152338	1.00E-06	4.80E-04	rs11076664	7.51E-39
16	RPL13	34	89617064	89643237	1.00E-06	4.80E-04	rs60724534	1.38E-35
16	SNORD68	32	89617837	89637909	1.00E-06	4.80E-04	rs60724534	1.38E-35
16	SPATA2L	53	89752764	89778121	1.00E-06	4.80E-04	rs258322	1.58E-36
16	SPATA33	112	89714151	89746866	1.00E-06	4.80E-04	rs35063026	3.70E-50
16	SPG7	213	89564795	89634176	1.00E-06	4.80E-04	rs11642231	2.33E-32
16	SPIRE2	200	89884906	89947727	1.00E-06	4.80E-04	rs34177108	8.52E-46
16	TCF25	149	89929993	89987792	1.00E-06	4.80E-04	rs1805007	1.12E-46
16	TUBB3	61	89978416	90012505	1.00E-06	4.80E-04	rs1805007	1.12E-46
16	URAHP	103	90096168	90124191	1.00E-06	4.80E-04	rs11076664	7.51E-39
16	VPS9D1	85	89763540	89797394	1.00E-06	4.80E-04	rs12925026	8.41E-44
16	VPS9D1-AS1	61	89768263	89794573	1.00E-06	4.80E-04	rs12925026	8.41E-44
16	ZNF276	181	89776775	89817332	1.00E-06	4.80E-04	rs12925026	8.41E-44
20	LOC101926888	36	32570293	32592173	1.00E-06	4.80E-04	rs6088372	2.05E-19
20	LOC101929746	55	32365178	32408905	1.00E-06	4.80E-04	rs117119427	1.94E-23
20	MAP1LC3A	45	33124687	33158149	1.00E-06	4.80E-04	rs6058079	8.29E-14
20	NCOA6	184	33292577	33423433	1.00E-06	4.80E-04	rs62211613	1.39E-25
20	PIGU	183	33138345	33275089	1.00E-06	4.80E-04	rs56238684	7.15E-27
20	ZNF341	115	32309565	32390075	1.00E-06	4.80E-04	rs111930714	9.13E-24
6	HLA-DRB6	400	32510489	32537779	2.00E-06	8.38E-04	rs113356426	4.36E-07
11	GRM5	899	88227743	88806846	2.00E-06	8.38E-04	rs7118021	1.93E-12
20	CHMP4B	70	32389109	32452173	2.00E-06	8.38E-04	rs117119427	1.94E-23
20	FAM83C	51	33863533	33890225	2.00E-06	8.38E-04	rs666006	1.46E-21
20	FAM83C-AS1	43	33863053	33883575	2.00E-06	8.38E-04	rs619865	9.95E-17
20	GGT7	76	33422522	33470661	2.00E-06	8.38E-04	rs11546155	6.81E-14
20	MMP24-AS1	52	33854135	33875960	2.00E-06	8.38E-04	rs2425039	8.79E-18

1	MDM4	150	204475506	204537248	3.00E-06	1.08E-03	rs12119098	2.51E-07
6	HLA-DQB1	691	32617240	32644466	3.00E-06	1.08E-03	rs4713570	8.06E-08
11	CCDC88B	56	64097689	64135006	3.00E-06	1.08E-03	rs61886926	1.40E-06
20	ACTL10	24	32244303	32266331	3.00E-06	1.08E-03	rs3213180	1.08E-18
20	DYNLRB1	42	33094188	33138762	3.00E-06	1.08E-03	rs137942933	2.35E-15
20	EIF2S2	54	32666114	32710085	3.00E-06	1.08E-03	rs6059662	1.46E-07
20	MIR4755	24	32626924	32646996	3.00E-06	1.08E-03	rs909884	1.45E-06
20	MMP24	123	33804538	33874804	3.00E-06	1.08E-03	rs2425025	1.50E-23
20	RALY	148	32571457	32680991	3.00E-06	1.08E-03	rs6059655	8.82E-34
6	C6orf52	26	10661650	10705030	4.00E-06	1.40E-03	rs9461031	9.49E-06
6	PSORS1C1	548	31072607	31117869	4.00E-06	1.40E-03	rs3094206	7.90E-06
11	RPS6KA4	72	64116624	64149687	5.00E-06	1.65E-03	rs61886926	1.40E-06
20	EIF6	55	33856708	33882619	5.00E-06	1.65E-03	rs619865	9.95E-17
20	HMGB3P1	33	33411377	33432265	5.00E-06	1.65E-03	rs62211621	1.78E-25
20	TP53INP2	50	33282147	33311237	5.00E-06	1.65E-03	rs6088579	1.89E-13
11	MIR1237	44	64126073	64146175	6.00E-06	1.92E-03	rs61886926	1.40E-06
20	SNTA1	59	31985762	32041698	6.00E-06	1.92E-03	rs293721	2.54E-21
6	C6orf15	308	31068999	31090332	7.00E-06	2.18E-03	rs3130977	1.60E-05
6	CDSN	335	31072864	31098252	7.00E-06	2.18E-03	rs3094206	7.90E-06
3	FOXP1	665	70993864	71643140	8.00E-06	2.46E-03	rs62247034	1.52E-11
20	E2F1	31	32253291	32284210	9.00E-06	2.73E-03	rs3213180	1.08E-18
1	CERS2	67	150927648	150957479	1.30E-05	3.84E-03	rs59988025	8.20E-06
20	NECAB3	36	32234892	32272264	1.30E-05	3.84E-03	rs3213180	1.08E-18
6	HCG23	214	32348286	32371468	1.50E-05	4.32E-03	rs115407878	1.49E-07
20	SLA2	53	35230923	35284619	1.50E-05	4.32E-03	rs55804368	3.26E-12
11	MIR7155	30	64099320	64119376	1.70E-05	4.84E-03	rs574835	6.92E-06
1	CTSS	109	150692671	150748433	2.00E-05	5.42E-03	rs41271951	2.55E-06
2	CTLA4	50	204722510	204748683	2.00E-05	5.42E-03	rs231775	1.63E-06
6	TRAM2-AS1	103	52431999	52458791	2.00E-05	5.42E-03	rs13208938	2.02E-05

20	CBFA2T2	130	32067927	32247837	2.00E-05	5.42E-03	rs17401449	5.19E-22
1	SETDB1	118	150888814	150947220	2.20E-05	5.90E-03	rs4970929	4.91E-06
1	ARNT	124	150772180	150859244	2.30E-05	6.09E-03	rs114845445	7.70E-06
6	PAK1IP1	15	10685187	10719970	2.50E-05	6.55E-03	rs9467242	1.39E-05
20	MIR499A	45	33568178	33588300	3.30E-05	8.55E-03	rs1885120	4.35E-24
2	CYP1B1	82	38284745	38313323	3.50E-05	8.96E-03	rs150795296	1.54E-05
1	FAM63A	78	150959300	150990854	3.60E-05	9.12E-03	rs12075317	3.80E-06
20	CDK5RAP1	73	31936644	31999375	4.10E-05	1.03E-02	rs293709	8.70E-19
6	MIR6891_2	773	31313000	31333093	4.20E-05	1.04E-02	rs145051174	5.01E-07
6	BTNL2	322	32352512	32384900	4.40E-05	1.07E-02	rs114275073	8.14E-08
20	MIR499B	45	33568202	33588275	4.40E-05	1.07E-02	rs1885120	4.35E-24
1	PRUNE	123	150970972	151018189	4.80E-05	1.15E-02	rs41310885	4.02E-06
1	ADAMTSL4	52	150511844	150543412	5.20E-05	1.24E-02	rs72700829	8.20E-06
1	HORMAD1	113	150660534	150703364	5.40E-05	1.27E-02	rs72702561	2.43E-06
15	MIR1272	48	65044585	65064714	5.70E-05	1.33E-02	rs111765533	4.24E-05
1	GOLPH3L	191	150608700	150679672	6.60E-05	1.52E-02	rs56057831	1.73E-05
6	HLA-B	869	31311648	31334989	7.10E-05	1.61E-02	rs145051174	5.01E-07
9	TYRP1	110	12683385	12720266	7.20E-05	1.61E-02	rs2762457	2.63E-06
11	PRDX5	15	64075559	64099295	7.20E-05	1.61E-02	rs646153	9.71E-06
15	TCF12	985	57200832	57590714	7.50E-05	1.66E-02	rs62022237	6.15E-06
6	CYP21A2_2	50	31996092	32019447	7.80E-05	1.71E-02	rs114645929	4.74E-04
8	TRPS1	404	116410723	116691255	9.90E-05	2.15E-02	rs9643105	1.66E-06
15	ZNF609	345	64781618	64988266	1.08E-04	2.33E-02	rs16948036	6.29E-05
1	MCL1	57	150537026	150562214	1.20E-04	2.56E-02	rs72700829	8.20E-06
11	ACRV1	65	125532228	125560793	1.23E-04	2.60E-02	rs34788353	9.06E-05
15	RBPMS2	122	65022094	65077770	1.28E-04	2.68E-02	rs111765533	4.24E-05
11	CHEK1	167	125485030	125556150	1.30E-04	2.70E-02	rs76978286	3.99E-05
1	CA14	32	150220217	150247480	1.42E-04	2.92E-02	rs113541041	1.33E-05
7	JAZF1	612	27860192	28230437	1.47E-04	3.00E-02	rs1635852	3.84E-06

20	ITCH	222	32941040	33109198	1.52E-04	3.07E-02	rs79777584	8.89E-26
15	OAZ2	45	64969772	65005462	1.56E-04	3.13E-02	rs11071804	1.27E-04
6	HCG27	435	31155536	31181745	1.65E-04	3.28E-02	rs147785311	9.39E-06
15	TRIP4	133	64670002	64757502	1.74E-04	3.43E-02	rs12101509	1.14E-04
4	PET112	259	152581808	152692175	1.77E-04	3.46E-02	rs3749562	1.69E-05
1	ADAMTSL4-AS1	67	150523370	150557028	1.81E-04	3.48E-02	rs72700829	8.20E-06
11	STT3A	178	125452689	125502654	1.81E-04	3.48E-02	rs11825214	6.56E-05
11	TRMT112	13	64073963	64095556	2.10E-04	3.97E-02	rs646153	9.71E-06
19	MIR8085	7	45251913	45271978	2.10E-04	3.97E-02	rs8100239	7.16E-05
11	KCNK4	9	64048792	64077503	2.32E-04	4.35E-02	rs2510066	1.68E-05
20	MYH7B	155	33533637	33600240	2.55E-04	4.74E-02	rs1885120	4.35E-24
11	GPR137	14	64041810	64066972	2.63E-04	4.85E-02	rs2510066	1.68E-05
1	PLEKHO1	50	150112169	150141825	2.88E-04	5.27E-02	rs55963510	1.99E-04
11	ESRRA	17	64062999	64094212	3.08E-04	5.59E-02	rs646153	9.71E-06
6	HLA-DRA	315	32397618	32422826	3.14E-04	5.65E-02	rs115921311	6.22E-06
2	RMDN2	493	38142461	38304285	3.24E-04	5.79E-02	rs1056837	1.86E-05
12	RAB21	100	72138657	72191150	3.33E-04	5.89E-02	rs12812659	6.05E-05
20	LINC00657	36	34623539	34648882	3.35E-04	5.89E-02	rs1204552	1.70E-08
11	BAD	21	64027299	64062176	3.58E-04	6.25E-02	rs2510066	1.68E-05
20	C20orf24	25	35224136	35250960	3.64E-04	6.31E-02	rs150260898	3.96E-12
17	TACO1	8	61668230	61695725	3.70E-04	6.35E-02	rs11871722	4.86E-04
11	PLCB3	42	64008994	64046924	3.72E-04	6.35E-02	rs12421615	9.76E-05
5	SQSTM1	148	179223387	179275077	3.75E-04	6.36E-02	rs248241	3.38E-05
6	HLA-DQA2	362	32699162	32724664	3.86E-04	6.49E-02	rs115507874	6.96E-08
19	LOC400684	70	32870955	32906445	3.91E-04	6.53E-02	rs10405382	6.70E-06
20	TRPC4AP	285	33580206	33690618	3.95E-04	6.55E-02	rs4911466	1.56E-23
17	MEOX1	112	41707757	41749262	4.15E-04	6.83E-02	rs6503462	1.25E-04
6	ZBTB12	57	31857393	31879769	4.21E-04	6.85E-02	rs115062572	2.60E-05
18	MEX3C	81	48690919	48734051	4.22E-04	6.85E-02	rs73438555	1.91E-04

18	LINC01254	128	10395129	10424367	4.28E-04	6.90E-02	rs206515	1.03E-04
1	BNIPL	69	150999028	151030076	4.38E-04	7.01E-02	rs12068631	6.06E-06
5	C5orf45	135	179254265	179295840	4.43E-04	7.03E-02	rs248241	3.38E-05
6	HLA-DQB2	205	32713874	32741330	4.45E-04	7.03E-02	rs115507874	6.96E-08
15	LOC101928227	88	38322593	38364541	4.54E-04	7.12E-02	rs61540283	4.22E-06
17	TRAF4	27	27061022	27087976	4.59E-04	7.15E-02	rs4313850	1.88E-04
20	PXMP4	70	32280549	32318136	4.66E-04	7.21E-02	rs17404047	5.70E-07
1	APH1A	25	150227798	150251609	4.82E-04	7.41E-02	rs11548267	1.74E-05
15	DUT	43	48613620	48645570	4.94E-04	7.54E-02	rs56308793	2.96E-04
11	CHRNA10	71	3676816	3702614	5.23E-04	7.93E-02	rs2741866	1.89E-04
6	PSORS1C2	229	31095310	31117127	5.35E-04	8.06E-02	rs114934621	5.09E-05
6	HUS1B	49	645938	666964	5.51E-04	8.25E-02	rs9405889	4.35E-08
20	EDEM2	156	33693159	33745161	5.69E-04	8.46E-02	rs7361656	2.59E-06
11	PPP1R14B	35	64001950	64024413	5.73E-04	8.46E-02	rs12421615	9.76E-05
1	SLAMF9	74	159911281	159934044	5.76E-04	8.46E-02	rs78643522	1.15E-04
17	FAM222B	161	27072995	27179857	6.01E-04	8.77E-02	rs4313850	1.88E-04
20	ACSS2	111	33452765	33525769	6.07E-04	8.80E-02	rs4911448	1.56E-12
1	CDC42SE1	46	151013446	151042125	6.27E-04	9.02E-02	rs11204765	1.75E-05
17	ERAL1	40	27172042	27198072	6.30E-04	9.02E-02	rs34901720	2.43E-04
11	IPO7	160	9396168	9479674	6.58E-04	9.36E-02	rs10840234	1.03E-04
17	MIR451B	35	27178388	27198456	6.66E-04	9.42E-02	rs34901720	2.43E-04
17	MIR451A	35	27178386	27198458	6.80E-04	9.44E-02	rs34901720	2.43E-04
9	SMU1	176	33031849	33086714	6.83E-04	9.44E-02	rs7872744	1.40E-04
11	ART1	163	3656360	3695646	6.83E-04	9.44E-02	rs2741866	1.89E-04
17	MIR144	35	27178550	27198636	6.84E-04	9.44E-02	rs34901720	2.43E-04
1	VPS45	127	150029349	150127505	6.92E-04	9.47E-02	rs72694942	2.31E-04
3	VENTXP7	100	21437217	21458177	6.94E-04	9.47E-02	rs442920	6.95E-04
15	FGF7	258	49705374	49789523	7.16E-04	9.66E-02	rs112326311	3.08E-04
20	BPIFB3	116	31633229	31671434	7.17E-04	9.66E-02	rs403598	1.53E-06

17	MIR4732	36	27178672	27198748	7.21E-04	9.66E-02	rs34901720	2.43E-04
1	CTSK	47	150758683	150790917	7.28E-04	9.70E-02	rs74857275	9.28E-06
10	ADO	40	64554515	64578239	7.45E-04	9.87E-02	rs7905654	1.08E-04
20	NDRG3	104	35270168	35384541	7.60E-04	9.96E-02	rs112710510	6.96E-12
6	TNXB_2	210	31998931	32087151	7.63E-04	9.96E-02	rs7774197	1.76E-04
19	DPY19L3	212	32886654	32986799	7.68E-04	9.96E-02	rs7253039	6.14E-06
11	WEE1	89	9585227	9621313	7.69E-04	9.96E-02	rs7939541	8.89E-05

p_fdr_bh=pvalue after false discovery rate (FDR) correction with Benjamini and Hochberg (1995) method; genes that reached the FDR controlled P<0.1 are presented. As 23,051 genes were tested, the P-value adjusted for Bonferroni correction was set as P<2.17x10-6 (0.05/23,051); genes that reached this Bonferroni-level of significance are highlighted in grey.

Supplementary Table 3. VEGAS2-Pathways association analysis top results

Pathway	empiricalP	Genes	bh_fdr_p
505578_GO:0042438_melanin_biosynthetic_process	1.00E-06	TRPC1_SLC45A2_TYRP1_TYR_PMEL_DCT_OCA2_MYO5A_MC1R_CTNS_ASIP_DDT	3.25E-03
491478_GO:0006582_melanin_metabolic_process	1.00E-06	TRPC1_SLC45A2_TYRP1_TYR_PMEL_DCT_OCA2_MYO5A_MC1R_CTNS_BCL2_ASIP_DDT	3.25E-03
751695_GO:0044550_secondary_metabolite_biosynthetic_process	1.00E-06	KMO_TRPC1_BDH2_SLC45A2_CYP11B2_TYRP1_TYR_PMEL_DCT_COQ6_OCA2_MYO5A_CYP1A2_MC1R_CTNS_ASIP_DDT	3.25E-03
498561_GO:0019748_secondary_metabolic_process	2.00E-06	AKR7A2_FMO1_KMO_CYP1B1_IL1B_UGT1A7_TRPC1_BDH2_CBR4_SLC45A2_PAM_DDC_PON3_AKR1B10_EPHX2_STAR_CYP11B2_TYRP1_AKR1C4_AS3MT_TYR_PMEL_ARL1_DCT_COQ6_OCA2_MYO5A_CYP1A2_MC1R_CTNS_BCL2_CYP2A6_ASIP_DDT_CYP2D6	4.87E-03
508487_GO:0046189_phenol-containing_compound_biosynthetic_process	4.00E-06	TGFB2_NR4A2_TRPC1_SNCA_HAND2_SLC6A3_SLC45A2_CRHR2_DDC_GPR37_TYRP1_GATA3_TPH1_TYR_PMEL_TPH2_PAH_DAO_DCT_GCH1_OCA2_CHRNA7_HDC_MYO5A_MC1R_CTNS_PNMT_INSM1_ASIP_DDT	7.79E-03
508446_GO:0046148_pigment_biosynthetic_process	6.00E-06	ATPIF1_UROD_PPOX_IBA57_SLC25A38_ALAS1_CPOX_TRPC1_GMPS_SOX2_PAICS_SLC45A2_TMEM14C_TYRP1_FXN_ALAD_PRTFDC1_COX15_UROS_TYR_HMBS_SLC11A2_PMEL_DCT_OCA2_ADAL_MYO5A_MC1R_CTNS_COX10_SHMT1_SLC25A39_NFE2L1_FECH_AP3D1_ASIP_ADA_GART_CECR1_DDT	9.74E-03
505580_GO:0042440_pigment_metabolic_process	4.60E-05	ATPIF1_UROD_PPOX_IBA57_UGT1A1_SLC25A38_ALAS1_CPOX_TRPC1_GMPS_SOX2_PPARGC1A_PAICS_BDH2_SLC45A2_TMEM14C_BLVRA_TYRP1_FXN_ALAD_AMBP_PRTFDC1_COX15_UROS_HPX_TYR_HMBS_SLC11A2_PMEL_DCT_OCA2_ADAL_MYO5A_HMOX2_MC1R_CTNS_COX10_SHMT1_SLC25A39_NFE2L1_FECH_BCL2_AP3D1_BLVRB_ASIP_ADA_GART_CECR1_DDT_HMOX1	6.40E-02

bh_fdr_p=pvalue after false discovery rate (FDR) correction with Benjamini and Hochberg (1995) method; pathways/gene-sets that reached the FDR controlled P<0.1 are presented. As 9,736 pathways/gene-sets were tested, the P-value adjusted for Bonferroni correction was set as P<5.14x10-6 (0.05/9,736); pathways/gene-sets that reached this Bonferroni-level of significance are highlighted in grey.

Supplementary Figure 1. Zoom plot of 16q24 locus

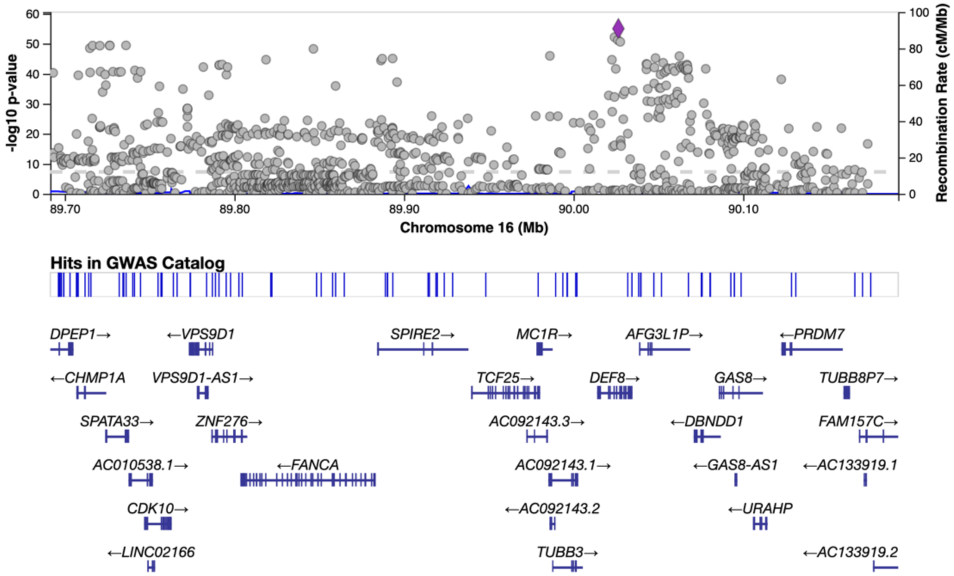

Supplementary Figure 2. Zoom plot of (a) 3p13, (b) 5p13, (c) 6p21, (d) 8q23, (e) 9p22, (f) 15q13, and (g) 20q11 loci

(a)

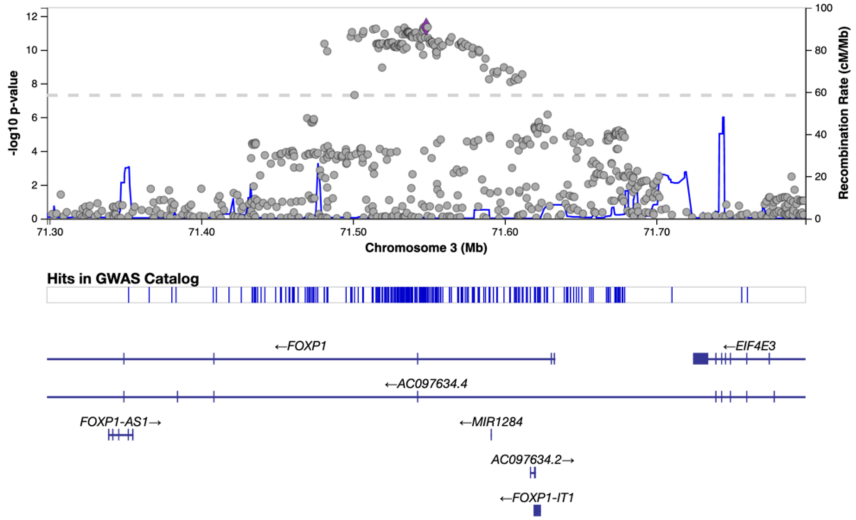

(b)

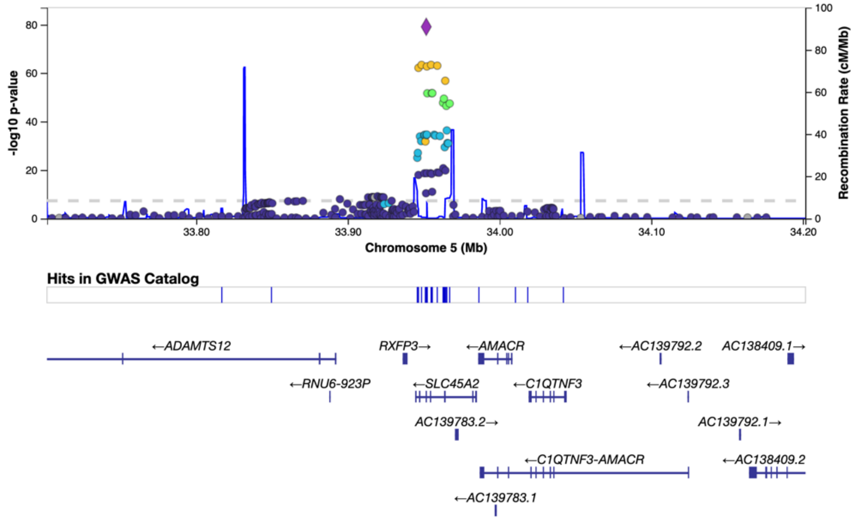

(c)

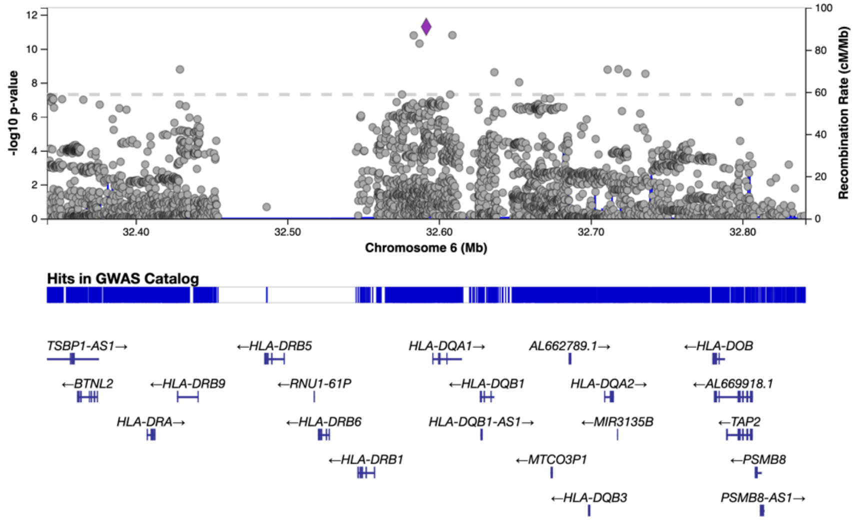

-log10 p-value
Recombination Rate (cM/Mb)
Chromosome 6 (Mb)
Hits in GWAS Catalog
TSBP1-AS1→
←HLA-DRB5
HLA-DQA1→
AL662789.1→
←HLA-DOB
←BTNL2
←HLA-DRB9
←RNU1-61P
←HLA-DQB1
HLA-DQA2→
←AL669918.1
HLA-DRA→
←HLA-DRB6
HLA-DQB1-AS1→
←MIR3135B
←TAP2
←HLA-DRB1
←MTCO3P1
←PSMB8
←HLA-DQB3
PSMB8-AS1→

(d)

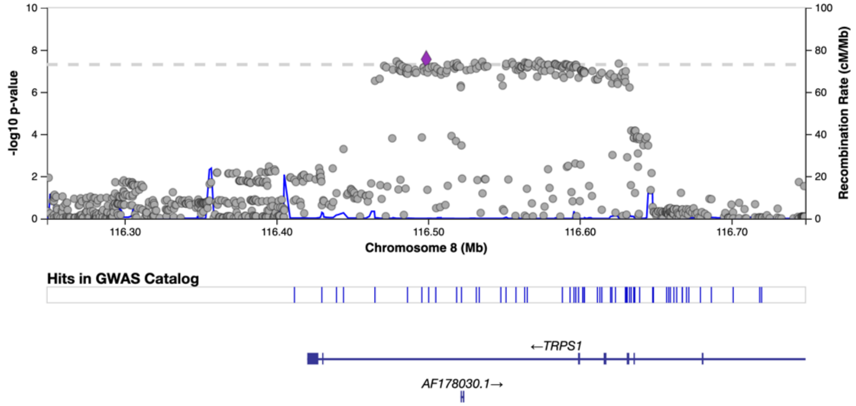

-log10 p-value
Recombination Rate (cM/Mb)
Chromosome 8 (Mb)
Hits in GWAS Catalog
←TRPS1
AF178030.1→

(e)

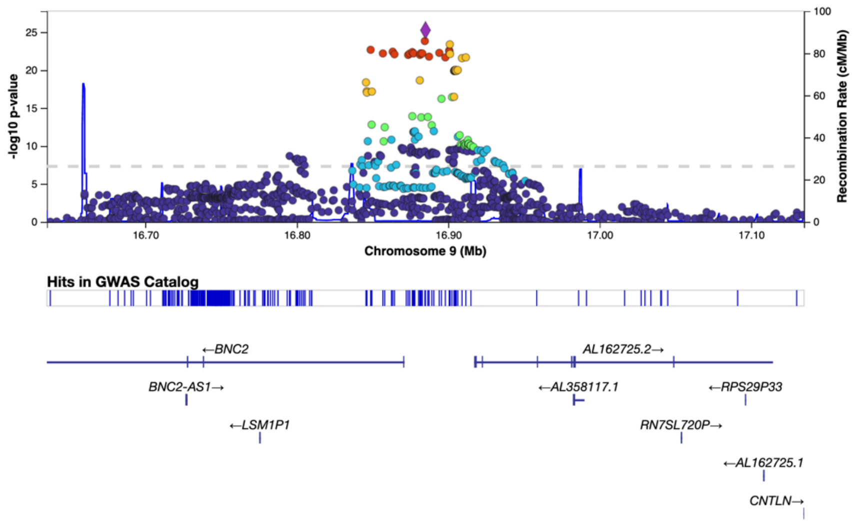
-log10 p-value
Recombination Rate (cM/Mb)
Chromosome 9 (Mb)
16.70
16.80
16.90
17.00
17.10
Hits in GWAS Catalog
←BNC2
AL162725.2→
BNC2-AS1→
←AL358117.1
←RPS29P33
←LSM1P1
RN7SL720P→
←AL162725.1
CNTLN→

(f)

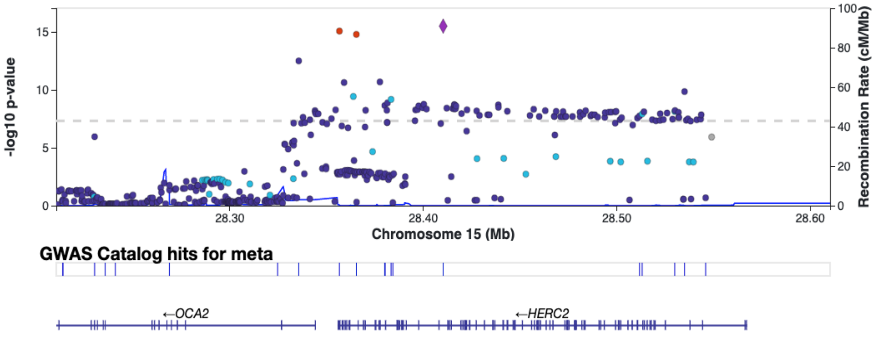
-log10 p-value
Recombination Rate (cM/Mb)
Chromosome 15 (Mb)
28.30
28.40
28.50
28.60
GWAS Catalog hits for meta
←OCA2
←HERC2

(g)

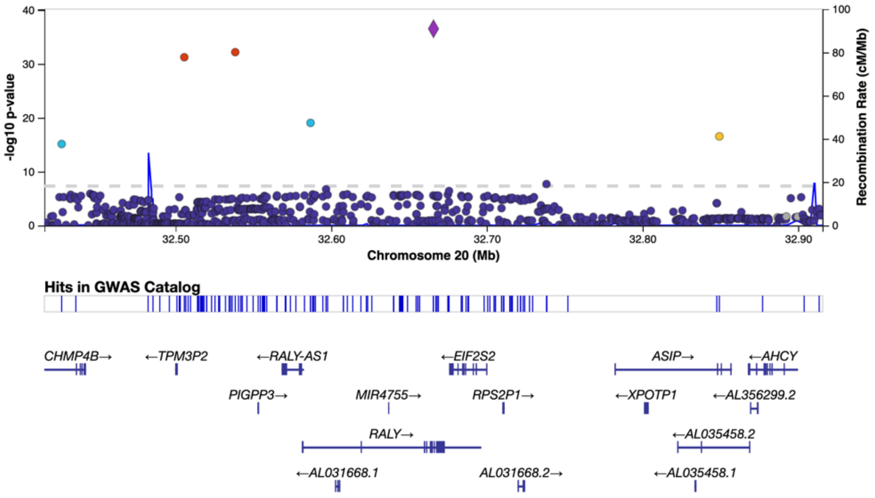

-log10 p-value
Recombination Rate (cM/Mb)
Chromosome 20 (Mb)
32.50
32.60
32.70
32.80
32.90
Hits in GWAS Catalog
CHMP4B→
←TPM3P2
←RALY-AS1
←EIF2S2
ASIP→
←AHCY
PIGPP3→
MIR4755→
RPS2P1→
←XPOTP1
←AL356299.2
RALY→
←AL035458.2
←AL031668.1
AL031668.2→
←AL035458.1
