# Supplementary material for: Genome-wide association study of actinic keratosis identifies new susceptibility loci implicated in pigmentation and immune regulation pathways
Source: Commun Biol. 2022 Apr 21;5:386. doi: 10.1038/s42003-022-03301-3 (PMC9023580; doi:10.1038/s42003-022-03301-3)
Supplement: Supplementary file 3 — Reporting Summary [file 42003_2022_3301_MOESM3_ESM.pdf]

nature portfolio

Corresponding author(s): Maryam M. Asgari

Last updated by author(s): Nov 3, 2021

Reporting Summary

Nature Portfolio wishes to improve the reproducibility of the work that we publish. This form provides structure for consistency and transparency in reporting. For further information on Nature Portfolio policies, see our Editorial Policies and the Editorial Policy Checklist.

Statistics

For all statistical analyses, confirm that the following items are present in the figure legend, table legend, main text, or Methods section.

n/a	Confirmed	
☐	☒	The exact sample size (*n*) for each experimental group/condition, given as a discrete number and unit of measurement
☐	☒	A statement on whether measurements were taken from distinct samples or whether the same sample was measured repeatedly
☐	☒	The statistical test(s) used AND whether they are one- or two-sided<br>*Only common tests should be described solely by name; describe more complex techniques in the Methods section.*
☐	☒	A description of all covariates tested
☐	☒	A description of any assumptions or corrections, such as tests of normality and adjustment for multiple comparisons
☐	☒	A full description of the statistical parameters including central tendency (e.g. means) or other basic estimates (e.g. regression coefficient) AND variation (e.g. standard deviation) or associated estimates of uncertainty (e.g. confidence intervals)
☐	☒	For null hypothesis testing, the test statistic (e.g. *F*, *t*, *r*) with confidence intervals, effect sizes, degrees of freedom and *P* value noted<br>*Give P values as exact values whenever suitable.*
☒	☐	For Bayesian analysis, information on the choice of priors and Markov chain Monte Carlo settings
☐	☒	For hierarchical and complex designs, identification of the appropriate level for tests and full reporting of outcomes
☒	☐	Estimates of effect sizes (e.g. Cohen's *d*, Pearson's *r*), indicating how they were calculated

Our web collection on statistics for biologists contains articles on many of the points above.

Software and code

Policy information about availability of computer code

Data collection	Genotype analysis, quality control, and imputation analysis were performed on samples from the GERA cohort using the following softwares: Affymetrix to perform genotype calling, quality control analysis, and sample or SNP filtering prior to downstream analysis. PLINK software v1.9 (www.cog-genomics.org/plink/1.9/) to perform addition QC analyses. Genotypes were then pre-phased with SHAPE-IT v2.5, and then imputed with IMPUTE2 v2.3.1. Genotype analysis, quality control, and imputation analysis were performed on samples from the MGB cohort using the following softwares: Illumina to perform genotyping. PLINK software v1.90 to perform addition QC analyses. Genotypes were then pre-phased with SHAPE-IT, and then imputed with Minimac3. All software programs employed are available for public use and no custom code was employed.
Data analysis	Eigenstrat v4.2 and PLINK software v1.90 were used to calculate the principal components (PCs) on GERA and MGB cohort, respectively. PLINK v1.9 (www.cog-genomics.org/plink/1.9/) was used to perform a logistic regression of the outcome and each SNP. Other statistic analyses and data management were performed using the R (version 3.6.2, http://www.R-project.org/) using functions from the default libraries. Versatile Gene-based Association Study-2 (VEGAS2) software was used to prioritize genes and biological pathways. The LD Hub web interface was used to calculate SNP-based heritability.

For manuscripts utilizing custom algorithms or software that are central to the research but not yet described in published literature, software must be made available to editors and reviewers. We strongly encourage code deposition in a community repository (e.g. GitHub). See the Nature Portfolio guidelines for submitting code & software for further information.

Data

Policy information about availability of data

All manuscripts must include a data availability statement. This statement should provide the following information, where applicable:

- Accession codes, unique identifiers, or web links for publicly available datasets
- A description of any restrictions on data availability
- For clinical datasets or third party data, please ensure that the statement adheres to our policy

The GERA genotype data are available upon application to the KP Research Bank (https://researchbank.kaiserpermanente.org/). A subset of the GERA cohort consented for public use can be found at NIH/dbGaP: phs000674.v3.p3.

Field-specific reporting

Please select the one below that is the best fit for your research. If you are not sure, read the appropriate sections before making your selection.

☒ Life sciences ☐ Behavioural & social sciences ☐ Ecological, evolutionary & environmental sciences

For a reference copy of the document with all sections, see nature.com/documents/nr-reporting-summary-flat.pdf

Life sciences study design

All studies must disclose on these points even when the disclosure is negative.

Sample size	In this study, we conducted a genome-wide association analyses, followed by meta-analysis, including 63,110 (16,352 cases and 46,758 controls) non-Hispanic white participants of the Kaiser Permanente Genetic Epidemiology Research on Adult Health and Aging (GERA) cohort and 29,130 (5,110 cases and 24,020 controls) Mass-General Brigham Biobank (MGB) cohort containing 21,462 cases and 70,778 controls. Rather than performing a power calculation, we collected the largest possible GWAS for actinic keratosis to date to identify novel risk loci. Those who were consented and genotyped in the Genetic Epidemiology Research on Aging and Mass General Brigham cohorts were included in the study. Cases in both cohorts were defined as participants who had a clinician-rendered actinic keratosis (AK) diagnosis (International Classification of Disease (ICD) diagnosis code version 9 of 702.0 and version 10 of L57.0) in the electronic health record. The control group included all participants without a relevant AK-ICD code.
Data exclusions	We applied standard quality control procedures for genotyped and imputed data, and the data did not meet the quality control steps were excluded. In GERA, genotype quality control (QC) procedures and imputation were conducted on an array-wise basis, after an updated genotyping algorithm with an advanced normalization step specifically for SNPs in batches not recommended or flagged by the outlier plate detector that has previously been done. Samples with sample call rate < 0.97 and SNPs with a call rate < 0.90 were filtered out. Imputation was done by array, and we additionally removed genetic markers with an imputation $R2 < 0.7$ and minor allele frequency (MA F) < 0.01. In MGB, an imputation $R2 < 0.8$, SNP call rates < 0.95, and MAF < 0.01 were excluded in the association analyses.
Replication	We sought to define the association in the Genetic Epidemiology Research on Aging discovery cohort and replicated the findings in the Mass General Brigham cohort.
Randomization	Samples were not randomized. This is a case-control study where cases were those with diagnosis of actinic keratosis, and controls were non-cases. We controlled the covariates by adjusting for age, sex, and ancestry principal components in the multivariable logistic models.
Blinding	Blinding was not relevant to our study, since participants were selected based on their diagnosis of actinic keratosis.

Reporting for specific materials, systems and methods

We require information from authors about some types of materials, experimental systems and methods used in many studies. Here, indicate whether each material, system or method listed is relevant to your study. If you are not sure if a list item applies to your research, read the appropriate section before selecting a response.

Materials & experimental systems

n/a	Involved in the study	
☒	☐	Antibodies
☒	☐	Eukaryotic cell lines
☒	☐	Palaeontology and archaeology
☒	☐	Animals and other organisms
☐	☒	Human research participants
☒	☐	Clinical data
☒	☐	Dual use research of concern

Methods

n/a	Involved in the study	
☒	☐	ChIP-seq
☒	☐	Flow cytometry
☒	☐	MRI-based neuroimaging

Human research participants

Policy information about studies involving human research participants

Population characteristics	The Genetic Epidemiology Research in Adult Health and Aging cohort consists of 110,266 adult men and women, 18 years and older, who are of non-Hispanic white, Hispanic/Latino, Asian or African American race-ethnicity. The Mass General Brigham cohort consists of 130,159 men and women, who are of non-Hispanic white, Hispanic/Latino, Asian or African American race-ethnicity.
Recruitment	The Resource for Genetic Epidemiology Research on Aging Cohort is the the Kaiser Permanente Research Program on Genes, Environment, and Health cohort consisted of about 140,000 individuals, and the participants provided self-reported information via the Research Program on Genes, Environment, and Health survey. The Mass General Brigham Biobank consisted of more than 120,000 individuals. They have provided consent to join and answered a detailed survey, provided saliva samples for extraction of DNA, and given broad consent for the use of their data in studies of health and disease.
Ethics oversight	The Institutional Review Boards at the Kaiser Foundation Research Institute and the Mass General Brigham Human Research Committee approved all study procedures.

Note that full information on the approval of the study protocol must also be provided in the manuscript.
